# Multiple observations of Bigfin Squid (*Magnapinna* sp.) in the Great Australian Bight reveal distribution patterns, morphological characteristics, and rarely seen behaviour

**DOI:** 10.1371/journal.pone.0241066

**Published:** 2020-11-11

**Authors:** Deborah Osterhage, Hugh MacIntosh, Franziska Althaus, Andrew Ross

**Affiliations:** 1 CSIRO Oceans and Atmosphere, Commonwealth Scientific and Industrial Research Organisation, Hobart, Tasmania, Australia; 2 Museums Victoria, Melbourne, Victoria, Australia; 3 CSIRO Energy, Commonwealth Scientific and Industrial Research Organisation, Australian Resources Research Centre, Kensington, Western Australia, Australia; Institut de recherche pour le developpement, FRANCE

## Abstract

One of the most remarkable groups of deep-sea squids is the Magnapinnidae, known for their large fins and strikingly long arm and tentacle filaments. Little is known of their biology and ecology as most specimens are damaged and juvenile, and in-situ sightings are sparse, numbering around a dozen globally. As part of a recent large-scale research programme in the Great Australian Bight, Remotely Operated Vehicles and a towed camera system were deployed in depths of 946–3258 m resulting in five *Magnapinna* sp. sightings. These represent the first records of Bigfin Squid in Australian waters, and more than double the known records from the southern hemisphere, bolstering a hypothesis of cosmopolitan distribution. As most previous observations have been of single *Magnapinna* squid these multiple sightings have been quite revealing, being found in close spatial and temporal proximity of each other. Morphological differences indicate each sighting is of an individual rather than multiple sightings of the same squid. In terms of morphology, previous in-situ measurements have been roughly based on nearby objects of known size, but this study used paired lasers visible on the body of a *Magnapinna* squid, providing a more accurate scaling of size. Squid of a juvenile size were also recorded and are confirmed to possess the long distal filaments which have thus far been mostly missing from specimens due to damage. We have described fine-scale habitat, in-situ colouration, and behavioural components including a horizontal example of the ‘elbow’ pose, and coiling of distal filaments: a behaviour not previously seen in squid. These sightings add to our knowledge of this elusive and intriguing genus, and reinforce the value of imagery as a tool in deep-sea squid research.

## Introduction

Deep-sea cephalopods are highly diverse and widespread yet often shrouded in mystery. Basic biological and ecological knowledge are lacking for many species and little baseline data exists, largely due to the inaccessibility of their vast yet little explored deep-sea environments [1,2]. Recent surveys in Australian waters, including those described in this study, provide an example of the importance of further deep-sea exploration, with high proportions of undescribed species and new species records found [3–7]. However, while increases in knowledge are expected as surveys expand into deeper waters, deep-sea cephalopod specimens are commonly damaged when collected by trawls, limiting insights from morphological examination [1,8]. The use of underwater imagery as a sampling tool in the deep-sea has revolutionised the study of such fragile fauna, enabling observations of live animals in-situ. For deep-sea cephalopods, this imagery has advanced knowledge of their natural morphology (e.g. posture, colour) [9], distribution [10], feeding and reproductive behaviours [11,12], and has at times revealed the unexpected and unique [11–15].

The study of Bigfin Squid, of the monotypic family Magnapinnidae, Vecchione and Young [16] provides a powerful example of the use of underwater imagery. The genus was first described from paralarvae and damaged juvenile specimens collected in epipelagic waters of the eastern Pacific [16]. Subsequent identification of *Magnapinna* sp. from video footage revealed the family’s most distinctive and peculiar feature: extremely long vermiform arm and tentacle filaments [14,17,18]. The imagery also proved *Magnapinna* to be a deep-sea squid, with broad distribution in bathyal and abyssal depths. A cosmopolitan distribution has been hypothesised, but to date only three sightings have been reported in the southern hemisphere [14,19,20]. Knowledge of *Magnapinna* remains limited, as collected specimens are damaged and/or juvenile, and only a dozen in-situ sightings have been recorded globally [14,17–21].

This paper reports on five *Magnapinna* sp. sightings made during a recent large-scale research programme in the Great Australian Bight (GAB), prior to which almost nothing was known of the GAB’s benthic deep-sea fauna [5,6,22,23]. These multiple sightings represent the first records of Bigfin Squid in Australian waters; this paper outlines the details and significance of the sightings and describes the observed morphology and behaviour of these distinctive squid.

## Methods

Offshore surveys using towed camera and Remotely Operated Vehicles (ROVs) were conducted as part of the Great Australian Bight Deepwater Marine Program, with the aim to characterise benthic diversity and geology of volcanic seamounts, submarine canyons and potential seep zones in southern Australia’s slope and abyssal waters [5,6].

**Fig 1:**
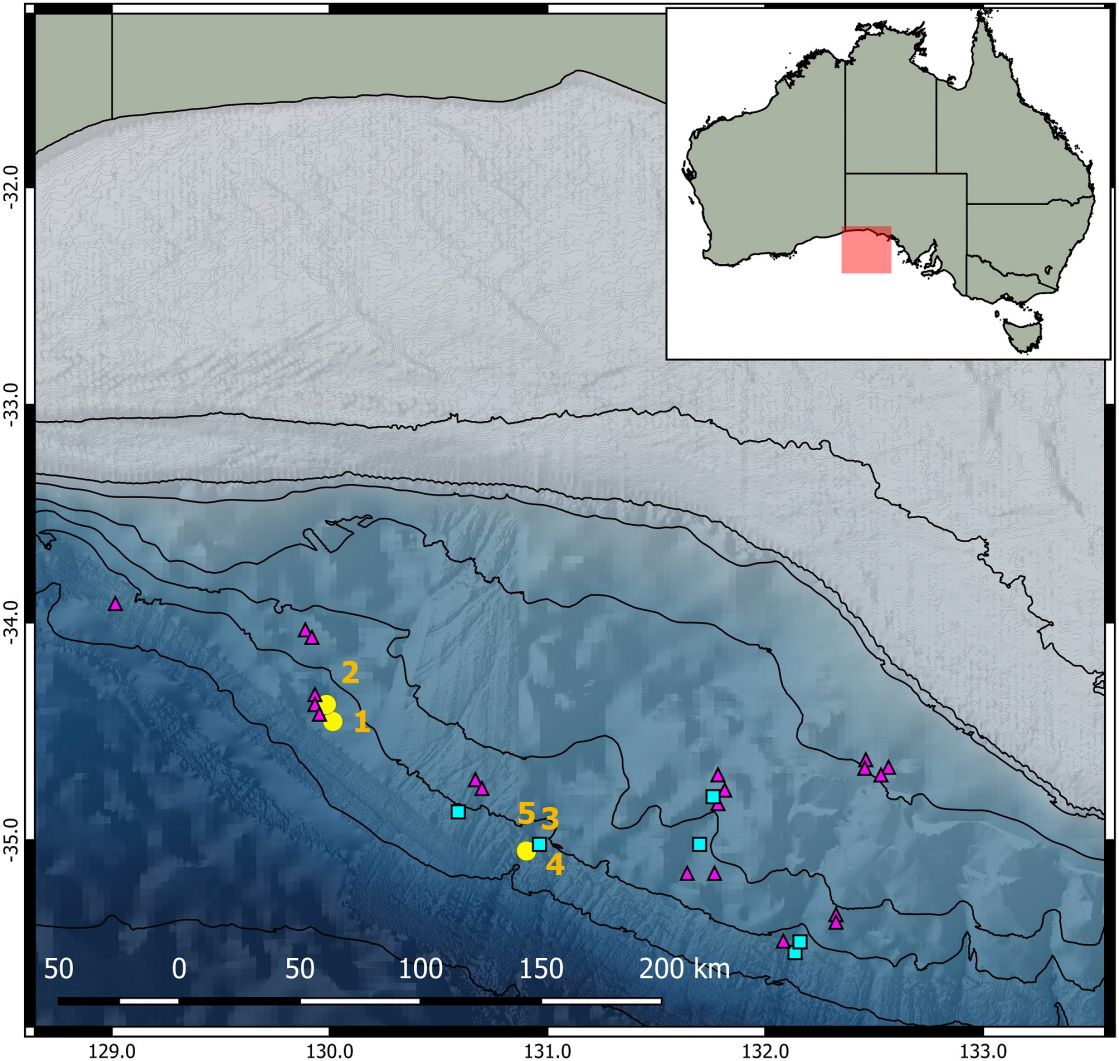
Observations of *Magnapinna* sp. in the Great Australian Bight. Observations of *Magnapinna* sp. (yellow circles) with sighting numbers, and the locations visually surveyed by towed camera (pink triangles) and Remotely Operated Vehicles (blue squares) in the Great Australian Bight (GAB). Bathymetric contours are 100, 200, 400, 1000, 1500, 2000, 3000 and 5000 m. Inset shows the location of the study area in the GAB.

Towed camera surveys were conducted in depths of 946–2400 m using the Marine National Facility deep towed camera system on the RV *Investigator*, with 22 benthic video transects undertaken in November 2015 (survey IN2015_C01) and 3 in April 2017 (survey IN2017_C01) (Fig 1). The camera system was equipped with a high definition video camera and a still image camera, both set at an oblique viewing angle, with paired lasers 100 mm apart for object sizing, and four remote controlled Deep Sea Power & Light SLS-3150 SeaLite Sphere floodlights with illumination in the daylight range (5000K-6500K) [24]. Still images were taken every 5 seconds. Tow speed was approximately 1 knot, with dynamic adjustment of the cable countering heave to maintain an altitude around 2 to 4 m above the seafloor. Geolocation of the camera system was achieved using an ultra-short baseline system and the ship’s global positioning system. Total survey time was 15.81 hrs, covering 46 km of linear transects, and approximating 218 km^2^ based on an average field of view width of 4.75 m [23]. Average field of view width was calculated by extrapolating paired laser measurements over 70 randomly selected images. All transect video was annotated for coarse habitat attributes, and initially 900 still images were randomly selected for fine-scale annotation of biota and habitat attributes (full details of annotation and survey methods can be found in [23]). Upon discovery of a *Magnapinna* squid during fine-scale annotation, all remaining still images (n = 10035) were checked for *Magnapinna* sp. and, where found, corresponding video footage was re-examined for the presence of *Magnapinna* sp.

ROV surveys were conducted at 7 sites in depths of 1332–3258 m during March 2017 (survey RE2017_C01) using two FCV 3000 work-class ROVs on the industry support vessel *REM Etive* (Fig 1). Each ROV was fitted with a high definition video camera, paired lasers (62 mm apart), and standard 4x LED lighting banks (2 x top and 2 x bottom) with variable illumination in the daylight range (5600K) and independent controllers [25]. One ROV had two additional lighting booms with variable and independent lighting controls. Grid transects were undertaken at each site, followed by subsequent specimen collections within the grids. During grid transects, the ROVs travelled at a speed of 2 knots and at an altitude of approximately 2–3 m above seafloor [23]. In response to previous *Magnapinna* sp. sightings from towed camera surveys, operators were instructed specifically to record all *Magnapinna* sp. observed during ROV operations. When sighted, still images were manually taken which allowed reference back to video time stamps. In total, 82 km of linear transects over a total area of 1 km^2^ were completed, and over 59.75 hrs of video collected using ROVs [23].

Measurements of morphology were taken with the image analysis software ImageJ [26], using paired lasers for scaling. Consistent with previous anatomical studies of *Magnapinna*, standard measurements included dorsal mantle length (DML, taken from the anterior mantle edge to the posterior junction of the fins), mantle width (MW), fin length (FL), fin width (FW), total arm length, and DML:FL [8,16,27]. To ensure accuracy, measurements were taken when the squid were as perpendicular as possible to the camera’s line of sight. In the two towed camera sightings, squid size were estimated from paired lasers on the adjacent seafloor, approximately ≤ 50 mm below the squid.

## Results

Five video observations of *Magnapinna* sp. were made in the GAB, with two sightings during the 2015 towed camera survey and three sightings during the 2017 ROV survey (Fig 1 and Table 1). Morphological measurements suggest that the five sightings represent five separate individuals (Table 2).

**Table 1:** Details of *Magnapinna* sp. observations in the Great Australian Bight.

Squid sighting	Sighting duration	Site	Gear	Time and Date (UTC)	Depth (m)	Gear altitude (m)	Latitude	Longitude
1	4 s	SZ08	TC	20:35, 15/11/2015	2178	1.5	-34.432	129.987
2	4 s	SZ08	TC	08:18, 16/11/2015	2110	2.3	-34.377	129.985
3	2 min 55 s	OR26a	ROV	20:06, 24/03/2017	3060	4.6	-35.049	130.905
4	37 s	OR26a	ROV	19:18, 25/03/2017	3002	6.5	-35.049	130.902
5	20 s	OR26a	ROV	21:13, 25/03/2017	3056	2.4	-35.050	130.904

Gear abbreviations: TC: Towed Camera; ROV: Remotely Operated Vehicle. Gear altitude is the Gear’s height above the seafloor recorded at the instance of first sighting for ROV, and at the taking of a still image for TC. See [23] for full Site details.

**Table 2:** Morphology measurements of *Magnapinna* sp. observed in the Great Australian Bight.

Squid sighting	Dorsal Mantle length (mm)	Mantle width (mm)	Fin length (mm)	Fin width (mm)	FL:DML ratio	Arm/tentacle length (mm)
1	116*	39*	87*	-	0.75	-
2	62*	11*	54*	66*	0.87	>1096
3	149	37	100	140	0.67	1680
4	-	-	-	-	0.95	-
5	-	-	-	-	0.79	-

Values marked with asterisk denote measurements estimated from paired lasers on adjacent seafloor. Arm/tentacle length represents length of longest arm/tentacle.

Although ROV and towed camera surveys covered a large area, *Magnapinna* sp. were only seen at two search sites: ‘SZ08’ situated on lower slope erosion channels (2015, towed camera), and ‘OR26a’ situated within a steeply sloping NNE-SSW oriented incised canyon (2017, ROV) (see [23] for full site details). Sightings by the towed camera system were 12 hours and 6 km apart and were brief (approx. 4 s), as the towed camera moved at a constant speed over the seafloor. The ROV sightings spanned 25 hours but were within 300 m of each other. ROV footage ranges from 20 s to 2 min 55 s, as the ROV was able to remain stationary or move with the squid.

ROV sightings occurred when the ROV was off-transect, that is, either when moving between transects or to locations for specimen collection. Generally, the ROV was flown at higher altitudes during these manoeuvres (up to 20 m off the seafloor), and at variable speeds.

### Sighting 1

**Fig 2:**
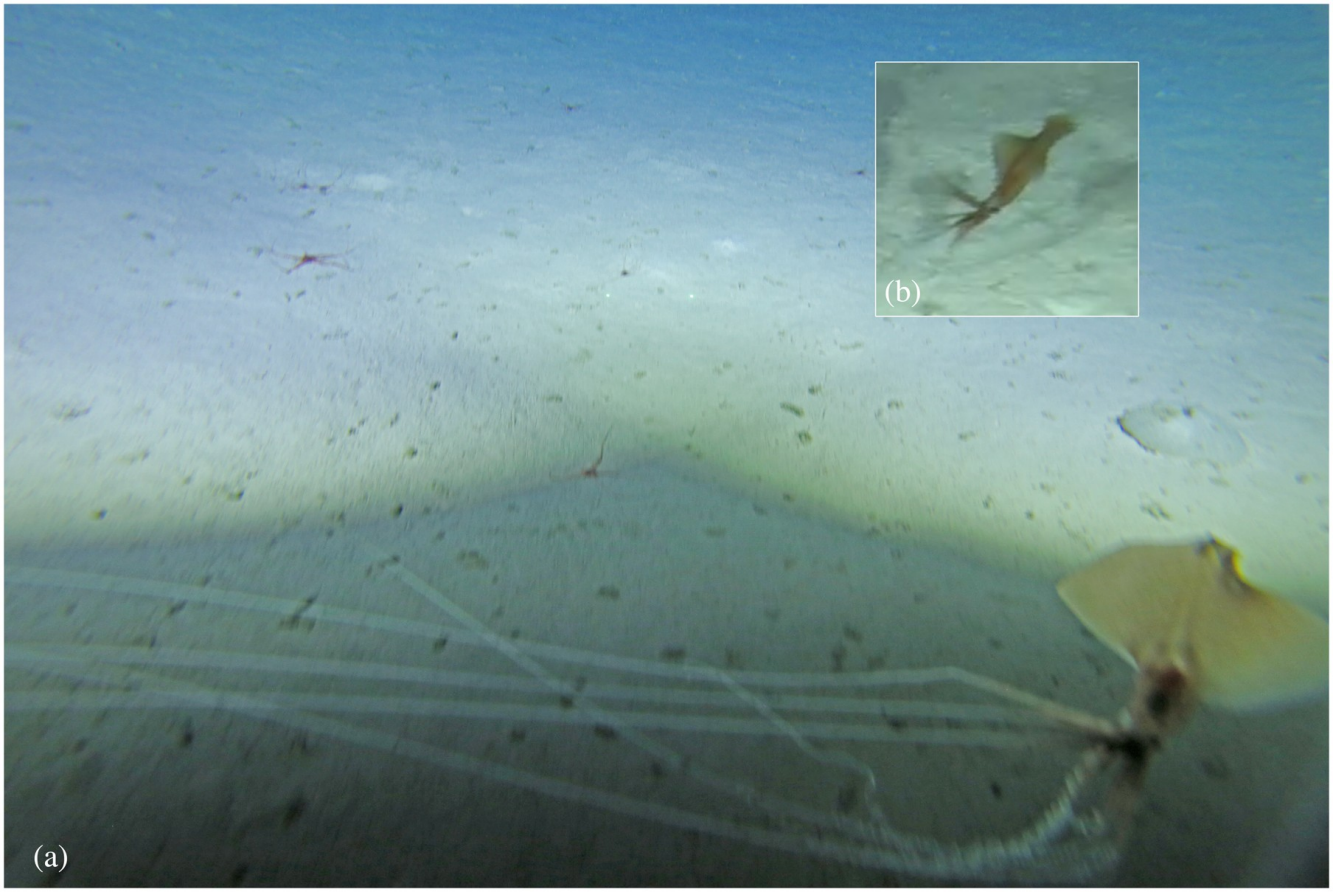
Sighting 1: Towed camera images of a *Magnapinna* squid at a depth of 2178 m. **(A)** A still image captured by towed camera. A *Magnapinna* squid was observed in the shadow of the towed camera system, just above the substrate with distal arm/tentacle filaments largely trailing parallel to the seafloor. Coiling of distal arm/tentacle filaments can be seen at their proximal ends. Image light levels have been adjusted for clarity, and distance between paired lasers seen on the seafloor is 100 mm. **(B)** A video image captured by towed camera. The squid is seen with proximal arms/tentacles spread, swimming upwards with undulating fins to a vertical position less than 100 mm from the seafloor. Colour is described from this image.

The first *Magnapinna* sp. sighting was by towed camera at a depth of 2178 m on 15 November 2015 (Fig 2, S1 Video). The squid was first observed in a horizontal position just above the seafloor, with proximal arms/tentacles spread and fins undulating. The squid appeared to be stationary evidenced by its unmoving position in relation to still objects on the seafloor. As the low-flying towed camera approached, the squid swam upwards (fin first) with undulating fins to a vertical position less than 100 mm from the seafloor. Distal arm/tentacle filaments were not visible in the video but were captured in the still image (Fig 2A), largely trailing parallel to the seafloor, and with approximately three distal filaments appearing coiled at their proximal ends (i.e at the junction with proximal arms/tentacles).

The colour of the squid appears a dark brown from the towed camera video, with mantle and proximal arms/tentacles slightly darker than the fins and head, and black eyes. An area of the dorsal mantle at and just below the fin junction appears dark reddish-brown (Fig 2B). The squid is underexposed in the still image, obscured by the shadow of the towed camera system. While the still image has been lightened for clarity, the colours themselves cannot be taken as representative, however comparative observations of shade can be made. The fine distal filaments which are only clearly visible in the still image are very light, appearing white; like the video, mantle and proximal arm/tentacles appear darker than the fins; and a dark circular mark is visible on the dorsal mantle.

Morphological measurements were made from video only, as the angle of the squid in the still image was not ideal, and its position on the edge of frame increased risk of lens distortion effects. Using paired lasers on the adjacent seafloor <50 mm below the squid, dimensions were estimated as 116 mm DML, 39 mm MW, 87 mm FL, and 0.75 FL:DML ratio (Table 2). Fin width could not be estimated as they were not fully extended in the video. The distal arm/tentacle filaments were only visible in the stills image: their length relative to DML could not be measured as their full length trailed outside the field of view, and a full count of arms/tentacles was not possible due to image quality.

The seafloor habitat was flat and consisted of fine light-coloured ooze with an overlay of possible biological origin (not gravel), and Lebensspuren including mounds and seastar imprints (as per [28]). Ophiuroids and an urchin test were visible in the immediate vicinity of the squid.

### Sighting 2

**Fig 3:**
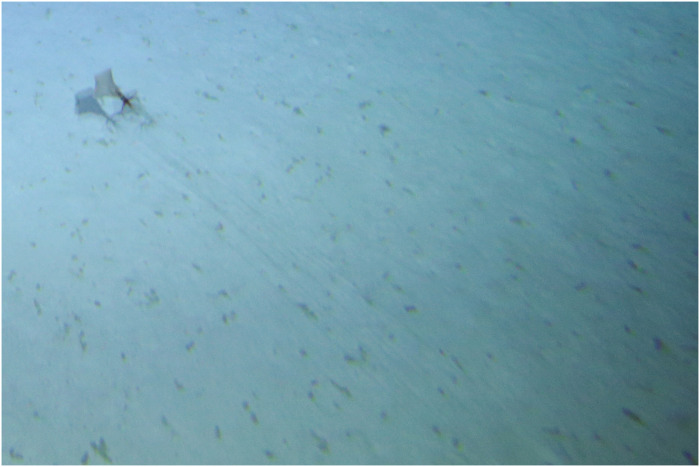
Sighting 2: Towed camera image of a *Magnapinna* squid at a depth of 2110. A small *Magnapinna* squid (62 mm DML) was seen in a horizontal position, parallel to and just above the seafloor. Its proximal arms/tentacles were spread outwards at an almost 90 degree angle to the body axis (causing the mantle and proximal arms/tentacles to appear as a cross-like shape in the image), and its distal arm/tentacle filaments streamed posteriorly, perpendicular to the proximal arms/tentacles and parallel to the seafloor (seen here as fine lines running diagonally from the squid to the bottom right of the image). The shadow of the *Magnapinna* squid can be seen below, duplicating the described posture. This image is an enlarged and cropped portion of a towed camera still image and light levels have been adjusted for clarity.

A second *Magnapinna* sp. observation was made on 16 November 2015 during a towed camera transect at 2110 m (Fig 3, S2 Video). The squid was observed in a horizontal position just above the seafloor: with proximal arms/tentacles held almost perpendicular to the body axis and distal arm/tentacle filaments trailing parallel to the seafloor. The squid appeared stationary in relation to Lebensspuren and a nearby urchin test, with slow undulations of the fins likely holding its position against current given that all distal filaments trailed straight behind. There was no apparent reaction to the towed camera as it passed quickly over the squid at an altitude of 2.3 m.

The video and still image have a blue-green cast so accurate colours descriptions were not possible, however, mantle and proximal arms/tentacles appear much darker than the fins and distal filaments.

This individual was smaller in size, with a DML of 62 mm and FW of 66 mm. The DML measurement was taken from video with scale estimated from paired lasers on the adjacent seafloor <50 mm below the squid. The remaining measurements were taken from the still image where detail of morphological features were clearer, and using the DML measurement from the video as reference scale. The arm/tentacle filaments were at least 1096 mm in length, but their full extent could not be measured as they extended beyond the camera frame (Table 2). A full count of arms/tentacles was not possible due to the small size of the squid and fineness of filaments, coupled with image quality.

Benthic habitat was similar to the first sighting, consisting of flat, fine, light coloured ooze with overlay of possible biological origin (not gravel) and Lebensspuren (including mounds and seastar imprints). Swimming sea cucumbers (*Enypniastes eximia*), a green urchin, and an urchin test with a crinoid atop were visible in the immediate vicinity of the squid.

### Sighting 3

**Fig 4:**
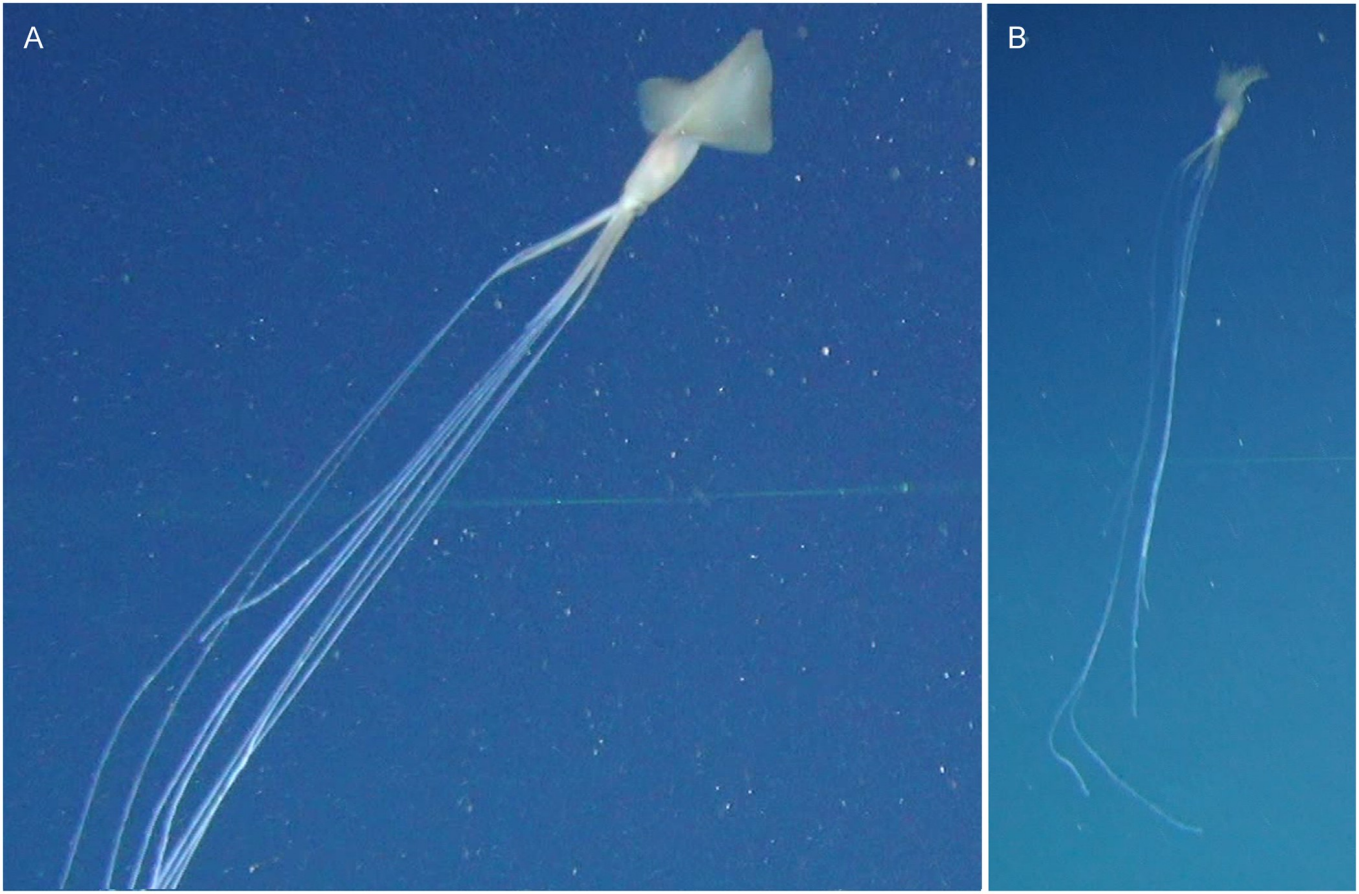
Sighting 3: ROV images of a *Magnapinna* squid at a depth of 3060 m. **(A)** Close view of the *Magnapinna* squid encountered 5.3 m above the seafloor with proximal arms/tentacles seen slightly opened and fins undulating slowly. **(B)** Full extent of arms/tentacles, the longest being approximately 1680 mm.

**Fig 5:**
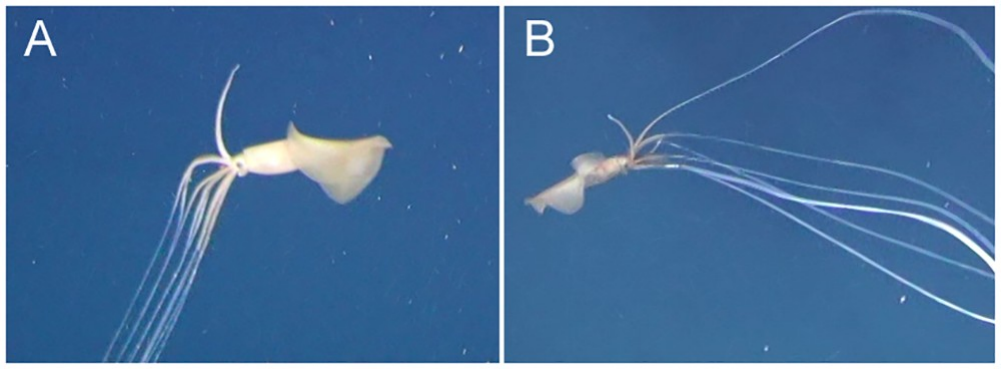
*Magnapinna* squid displaying raised arm behaviour. *Magnapinna* squid from **(A)** Sighting 3 and **(B)** Sighting 4 exhibited a postural behaviour, in which a single arm/tentacle (without filament) was raised perpendicular to the anterior-posterior body axis while the squid moved upwards and into a horizontal posture.

A third *Magnapinna* sp. sighting was made by ROV on 24 March 2017, at a depth of 3060 m (Fig 4, S3 Video). The ROV was at an altitude of 4.6 m, and the squid was encountered well below the ROV in an oblique position, with its proximal arms/tentacles held slightly opened at angles ranging from approximately 10–75 degrees. Its fins were undulating and distal arm/tentacle filaments trailed passively beneath with some disturbance from ROV thruster turbulence, particularly at the distal ends. After approximately 1 min 11 seconds the squid suddenly changed position, moving upwards and anteriorly to a horizontal position, whilst raising a single arm/tentacle. The raised arm/tentacle was without filament and held perpendicular to the anterior-posterior body axis (Fig 5A). Fin flapping appeared to pause momentarily during the upward movement. No expansion of mantle or discharge from the funnel was seen, however lack of detail caused by overexposure made such observations difficult. The raised arm/tentacle lowered as the squid swam away in a horizontal position with strong fin flapping. The observation lasted 2 min 55s, during which time the squid slowly increased altitude above the seafloor to approximately 9.4 m.

Representative colour descriptions were not possible from the majority of video footage of Sighting 3 as there was often a blue cast, or overexposure which caused loss of detail. However, Fig 4A shows the squid pale in colour, with a pinkish tinge at and below the fin junction, a dark eye, light brown fins, and white distal arm/tentacle filaments. The mantle and fins appear slightly translucent.

Paired lasers visible on the arms and the upright position of the body allowed for accurate estimation of morphology measurements: 149 mm DML, 37 mm MW, 100 mm FL, and 140 mm FW. Distal arm/tentacle filaments were not of a uniform length, ranging from approximately 536 mm to 1626 mm. One arm/tentacle lacked a distal filament.

Flat, light coloured ooze with Lebensspuren such as mounds were visible on the seafloor at this altitude, and marine snow and some zooplankton were visible in the water column.

### Sighting 4

**Fig 6:**
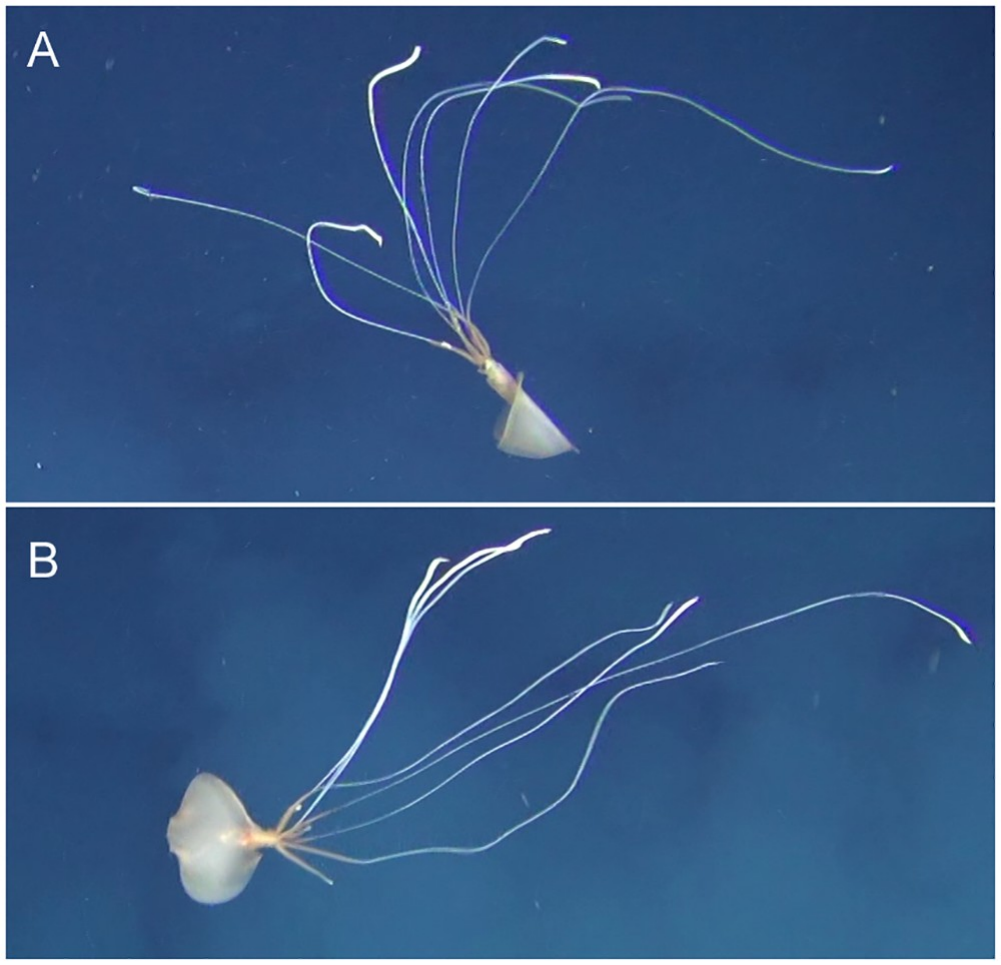
Sighting 4: ROV images of a *Magnapinna* squid at a depth of 3002 m. **(A)** Lateral view of *Magnapinna* squid encountered at a depth of 3002 m. **(B)** The *Magnapinna* squid swimming horizontally with rapid fin flapping in water affected by ROV thruster turbulence. The squid was missing three distal arm/tentacle filaments.

A fourth *Magnapinna* sp. sighting was made on 25 March 2017 at a depth of 3002 m whilst the ROV was 6.5 m above the seafloor (Fig 6, S4 Video). The squid appeared affected by water turbulence from ROV thrusters which also stirred up the sediment below. The squid entered the camera’s field of view, swimming with rapid fin flapping in a largely horizontal to slightly oblique position. After approximately 14 s the squid raised up an arm/tentacle (without distal filament) perpendicular to the anterior-posterior body axis, with its body becoming more precisely horizontal in position (Fig 5B). The squid moved upward during this manoeuvre, but it is unclear whether the upward movement was caused by turbulence. The squid became further affected by turbulence, spinning 180 degrees, then continued to swim away with fin flapping, one arm/tentacle raised, and distal filaments trailing passively under the effect of ROV turbulence. The encounter was brief, with the squid leaving the field of view after approximately 37 s.

Overall, the squid appeared orange to orange-brown, with fins slightly translucent and paler, proximal arms/tentacles slightly darker, distal arm/tentacle filaments white, and eye dark. A patch of darker orange could be seen at times on the dorsal mantle at and below the fin junction.

Only seven arms/tentacles of the squid possessed distal filaments. Distal arm/tentacle filament lengths were uneven but not to as great an extent as sighting 3, with the shortest being approximately 45% as long that of the longest. The paired lasers were not turned on at this time precluding morphological measurements, however a FL:DML ratio of 0.95 could be established.

The seafloor habitat below consisted of light-coloured fine ooze with sparse cobble overlay and Lebensspuren including mounds and pits. Nearby were rough low rock outcrops overlayed by fine ooze sediment. Swimming sea cucumbers (*Enypniastes eximia*) and marine snow were visible in the water column.

### Sighting 5

**Fig 7:**
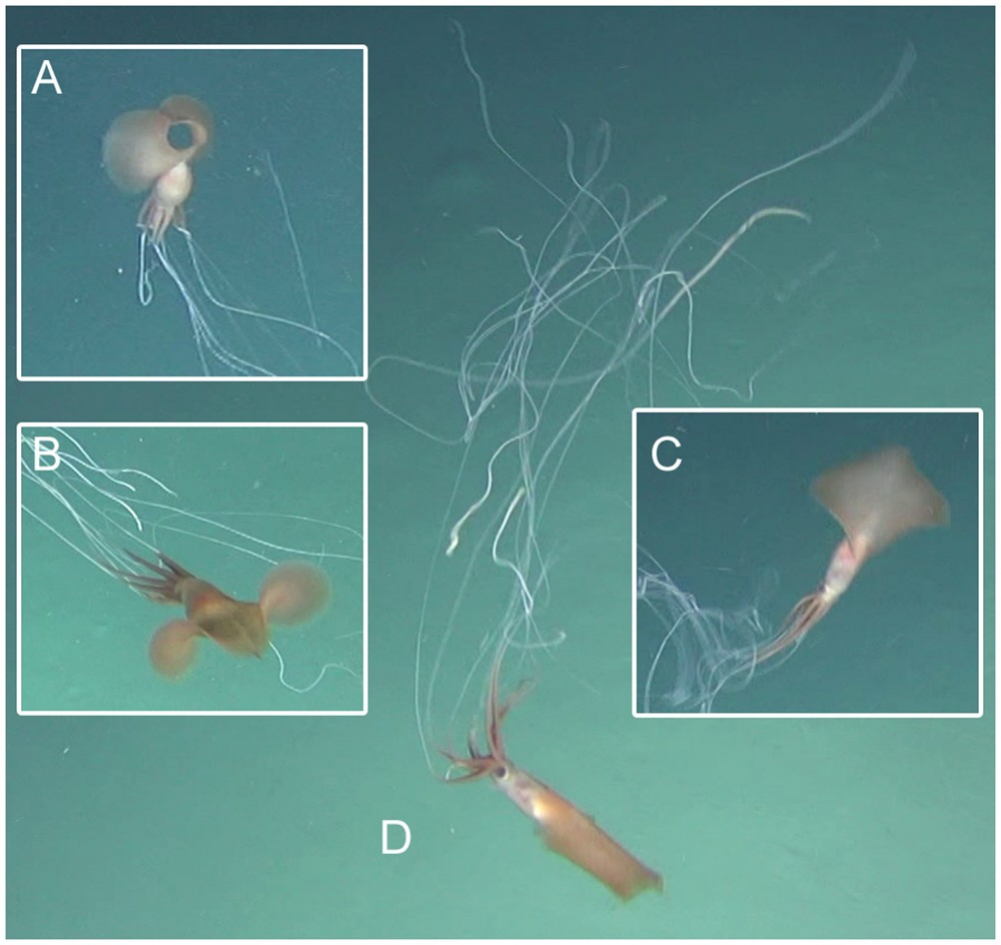
Sighting 5: ROV images of a *Magnapinna* squid at a depth of 3056 m. The *Magnapinna* squid was greatly affected by turbulence from ROV thrusters during this brief observation. Several colour descriptions were necessary for this squid. **(A)** Ventral view of the *Magnapinna* squid showing pale mantle and proximal arms/tentacles. **(B)** Dorsal view showing dark orange-brown mantle and proximal arms/tentacles. **(C)** Dorsal view showing a pale pink-brown mantle and light brown proximal arms/tentacles. **(D)** Lateral view showing a pale mantle and dark orange-brown proximal arms/tentacles.

A final, brief sighting was made on 25 March 2017 at a depth of 3056 m when the ROV was at a lower altitude of 2.3 m above the seafloor. The observed *Magnapinna* squid swam with strong fin flapping in an oblique to horizontal position with closed proximal arms/tentacles, and trailing distal arm/tentacle filaments that were affected by turbulence. The squid then became caught in the ROV’s thruster turbulence, spinning in a vortex current before leaving the camera’s field of view (Fig 7, S5 Video).

Rapidly changing viewing angles made colour description challenging for this sighting. An early ventral view revealed a pale mantle and proximal arms/tentacles, with fins a darker brown (Fig 7A). As the squid moved toward the camera in a horizontal swimming position, the dorsal mantle and proximal arms/tentacles appeared a dark orange-brown, with the fins and head slightly lighter (Fig 7B). The squid spun in a vortex current revealing another dorsal view, this time in an oblique vertical position, with mantle a pale pink-brown, an area of brighter pink-orange around the fin junction, a pale head, dark eyes, and fins and proximal arms/tentacles of a light brown (Fig 7C). Just before the squid left the camera’s field of view, a lateral view showed a pale mantle with an area of brighter yellow and orange at and below the fin junction, a black eye, and orange-brown fins and slightly darker proximal arms/tentacles (Fig 7D). The distal arm/tentacle filaments remained white throughout.

Scale could not be established due to poor visibility of the paired lasers, but a FL:DML ratio of 0.79 was measured (Table 2). The squid possessed at least 9 distal arm/tentacle filaments.

The seafloor below consisted of light-coloured fine ooze with some Lebensspuren including mounds and pits. Marine snow and some zooplankton were visible in the water column.

## Discussion

### Significance of sightings

These sightings represent the first records of *Magnapinna* squid in Australian waters, and they more than double the known records from the southern hemisphere [14,20]. While identification to species is not possible based solely on imagery, the morphology of the observed squids, with characteristic large fins and extremely long distal arm/tentacle filaments, clearly corresponds to the genus *Magnapinna* [29].

Five sightings in the Great Australian Bight is a considerable number, given previous observations of the family over 30 years total around a dozen globally [14,17,18,19,20,21]. Comparing the rate of *Magnapinna* squid sightings from previous surveys is difficult as relevant survey details are often not readily available. Where found, video hours are a common metric of unit effort, with 5 *Magnapinna* squid sighted in 75 hours of this study compared to 1 in 280 hours [14,30], 1 in 100 hours [18,31], 2 in 80 hours (not confirmed as individuals) [18,32], and 1 in 57 hours [19,33]. These numbers are suggestive of a higher than average rate of *Magnapinna* squid sightings in the GAB region, however should be interpreted with caution given the likelihood of variabilities in survey methods (e.g. underwater vehicle speeds).

It could be suggested the high number of GAB sightings is influenced by sampling bias, as all towed camera images were searched specifically for *Magnapinna* sp., and ROV operators recorded all sightings including those off transect. However, the majority of previous records have been from manned submersibles (n = 6) and commercial oil and gas ROVs (n = 4) during which all sightings, rather than randomly selected or those ‘on-transect’, would have been similarly recorded [14,19].

Underwater visual surveys can be subject to observer bias in which results, particularly in relation to rare species, may differ due to observer experience or interpretation [34–37]. Simple hierarchical classification schemes using consistent identifiers have been found to reduce observer variation (e.g. CATAMI) and although these schemes annotate at a broad scale, they allow the opportunity for revisiting by specialised taxonomists [38,39]. Using the methods undertaken in the GAB surveys, the *Magnapinna* sp. seen in our study would have been annotated as “Cephalopods: Squid”. However, the observer identified the genus of the squid and understood the rarity of the initial sighting, prompting further investigation. It is not known whether the outcome would have differed with another observer, and to date there have been no published studies by specialised taxonomists based on imagery of deep-sea squid in Australia. The peculiarity and distinctiveness of *Magnapinna* squid has meant that even incidental sightings from hydrocarbon well operations have been reported, but such peculiarity does not guarantee that generalist annotators would recognise the interest in its reporting [21]. It may be that imagery of *Magnapinna* squid are present in already surveyed areas, both in Australia and around the world, but have not yet come to light due to lack of identification at the appropriate taxonomic scale.

Whether the comparatively high number of *Magnapinna* squid sighted in Australia’s GAB equates to a *Magnapinna* sp. hotspot remains to be seen.

### Distribution pattern within the GAB

The Great Australian Bight survey spanned nearly 350 km of the GAB slope with over 75 hours of video recorded, however *Magnapinna* sp. sightings were limited to two sites where they were found clustered in close spatial and temporal proximity, with towed camera sightings being 12 hours and 6 km apart and ROV sightings spanning 25 hours and being within 300 m of one another (Fig 1, Table 1). Most previous reports have been of single sightings, with the exception of two in the Colombian Caribbean during exploratory hydrocarbon surveys (27 km and 2 months apart) [21], and two in the Eastern Atlantic (40 mins apart during same dive) [14]. The Eastern Atlantic observations were of similar close spatiotemporal proximity to those of the GAB, but could not be distinguished as individual *Magnapinna* squid, as opposed to multiple sightings of the same squid. Size and morphological differences seen in our study strongly suggest each of these clustered observations is of an individual *Magnapinna* squid (Table 2). Clustered mobile fauna has been previously reported in the deep-sea (e.g. macrourid fishes), with such clustering often associated with specific environmental needs and/or increased reproductive opportunities [40,41].

Fine-scale descriptions of habitat where previous *Magnapinna* sp. sightings have been made are limited, with one [18] reported above a “sedimentary seafloor” during a survey of a transform margin. All *Magnapinna* sp. sightings in the GAB were made in areas of predominantly soft sediment, in terrain of lower-slope erosion channels, and upper section of submarine canyon, between 2000 and 3000 m depth (Fig 1). Submarine canyons and similar incised features often support high productivity and diversity in the deep-sea, and these locations may reflect habitat preference of *Magnapinna* sp. [42]. Further fine-scale reporting of habitat in future sightings will assist in determining any patterns of habitat preference by *Magnapinna* squid.

### Morphological characteristics

#### Size

Morphology measurements estimated during Sighting 3 are the first known from paired lasers viewed directly on a *Magnapinna* squid, with previous in-situ measurements based on comparison to nearby objects of known size e.g. submersible components [18,20,21]. The total length (DML + longest arm/tentacle length) of the *Magnapinna* squid in Sighting 3 (~1830 mm) is likely longer than those measured in the southern hemisphere: >1500 mm total length [19], and <100 mm DML [20]; and shorter than those measured in the northern hemisphere (n = 6) ranging from 2250 to 7000 m in total length [14,21]. Two other *Magnapinna* squid were measured using paired lasers on the adjacent seafloor ~50mm below, and are amongst the smallest in-situ *Magnapinna* sp. recorded with DMLs of 62 mm and 116 mm. These squid are within the range of DML measured in juvenile *Magnapinna* sp. specimens, which are mostly missing distal filaments due to damage [29]. Our imagery confirms that squid of this size do possess long distal filaments, with those seen in Sighting 2 measuring >17 times the DML.

#### Colour

In-situ colours of *Magnapinna* squid are not often reported, though [18] describes the mantle as “brownish” and [14] and [18] note the filaments are white or comparatively lighter in colour. Generally, the *Magnapinna* squid observed in the GAB were of brown, orange, and pink hues, ranging from pale to dark tones; distal arm/tentacle filaments were white, eyes dark, and fins and mantle often slightly translucent. An area of orange/pink was also commonly seen where fins meet mantle. This may represent an internal organ seen through the translucent mantle, possibly corresponding with an orange digestive gland described in *M*. *atlantica* [29]. Variation in colouration seemed to exist between squid (e.g. Sighting 1, Fig 2 is a much darker brown, though this may represent differences between gear), and within sightings (e.g. Sighting 5, Fig 7A–7D). The changes seen in Sighting 5 may represent chromatic colour change, however rapidly changing viewing angles caused by ROV thruster turbulence makes it difficult to say this with certainty. Whilst chromatic colour changes in the dark deep-sea environment may seem counterintuitive, recent studies have found deep-sea squid capable of a wide variety of chromatic colour changes, and most specimens of *Magnapinna* sp. possess abundant chromatophores [10,20,29,43]. In-situ colour change has not been reported before for *Magnapinna* sp., however would certainly be of interest to note in future sightings.

### Behaviour

#### Locomotion and postural components

Recent studies have found deep-sea squid to have an abundant array of behavioural components, including locomotive and postural, that are comparable to or exceed those recognised in shallow water cephalopods [10,43]. Opportunity to observe behavioural components in this study was limited, with <4 minutes of video footage. Locomotion was largely as described previously in [17] with sinusoidal undulations and flapping of fins. Two *Magnapinna* squid (Sightings 3 and 4) exhibited a postural behaviour, in which a single arm/tentacle (without filament) was raised perpendicular to the anterior-posterior body axis while the squid moved upwards and into a horizontal posture. Squid 4 was affected by turbulence during this manoeuvre and it was unclear whether the upward movement was a result of turbulence, but for Squid 3 the position change was sudden and fin flapping seemed to pause momentarily during the upward movement. This may suggest the use of jetting, however overexposure obscured detail in the squid’s body and no discharges were observed from the funnel. The functional role of this postural behaviour is unknown, however it does share similarities to variations of the Dorsal Arm Curl seen in the deep-sea squid *Octopoteuthis deletron* and other equivalent postures in deep and shallow water squid, though these involve raising more than one arm [10].

#### Horizontal ‘elbow’ pose

One of the most distinctive behavioural characteristics of *Magnapinna* squid is the commonly seen ‘elbow’ pose: a vertical or oblique posture in which proximal arms/tentacles are spread outwards, and distal arm/tentacle filaments dangle downwards at sharp angles (almost 90 degrees) toward the seafloor [14,17,19,21]. Recent video observations of deep-sea chiroteuthid squid have revealed similarities in behaviour: some mastigoteuthids orientate vertically and dangle long whip-like tentacles in a “tuning fork” pose, and some *Chiroteuthis* squid dangle long tentacles at sharp right angles to their arms [43–45]. These squid are assumed to be “fishing”, with some *Mastioteuthis* squid capturing small plankters with minute, sticky suckers [44]. The purpose of the ‘elbow’ pose in *Magnapinna* sp. is not known, though the highly adhesive nature of their extremely long arm and tentacle filaments has led some to postulate a similar feeding function [1].

The ‘elbow’ pose seen in Sighting 2 (Fig 3) was unusual in that the squid postured horizontally rather than upright in relation to the seafloor. This horizontal ‘elbow’ pose is rarely seen; only previously reported by [18] in which the squid was observed mid-water. Sighting 2 shows a *Magnapinna* sp. maintaining this pose just centimetres above the seafloor with distal arm/tentacle filaments trailing approximately parallel to each other and the seafloor. Similarly, Sighting 1 was horizontal and just above seafloor when first observed, but the near 90 degree angle of the ‘elbow’ pose could not be confirmed for all arms/tentacles before its disturbance by the towed camera system (Fig 2). Whether such close proximity to the seafloor is for the purpose of ‘fishing’ for demersal prey or avoiding pelagic predators (particularly as these two squid appear juvenile in size) remains to be seen.

Proximal arms/tentacles were also seen opened at smaller angles, but no other sharp ‘elbow’ positions were observed, likely because of disturbance caused by ROV thrusters. Sighting 2 was the only squid in an ‘elbow’ pose, and the only squid with no reaction to the camera system; presumably because the towed camera passed quickly and high above. Other studies recording ‘elbow’ poses have seen little initial reaction to deep-sea vehicles, but observed reactions when turbulence was encountered [14]. From our observations, the effect of turbulence on distal filaments can be substantial (e.g. Fig 7), suggesting the filaments are poorly muscularised and may be reliant on water movement in the case of horizontal ‘elbow’ poses, or a lack thereof in vertical ‘elbow’ poses, to maintain the characteristic parallel extension of filaments seen in such ‘elbow’ poses.

#### Filament coiling

A towed camera image from Sighting 1 appears to show coiling of several arm/tentacle filaments at their proximal ends; a behaviour not previously seen in squids (Fig 2). Comparative examples within the Cephalopoda are rare, as to date filament coiling has only been reported in the distantly related cephalopod *Vampyroteuthis infernalis* (Order: Vampyromorpha), which extends filaments up to eight times its body length, retracting them by helical coiling [12]. It has been previously noted that *Magnapinna* squid filaments are retractile [14,17], and whilst there are obvious differences between the filamentous appendages of *V*. *infernalis* and *Magnapinna* squid (e.g. *V*. *infernalis* has two modified arm filaments covered in fine hairs for collection of detrital food; *Magnapinna* squid have filaments extending from all arms and tentacles with numerous minute suckers), it may be that coiling behaviour represents an efficient biomechanical solution to the retraction of such long, thin filaments [12,16]. Further in-situ imagery of filament coiling, particularly of a high quality, or the collection of an intact specimen would assist in the interpretation of this behaviour in *Magnapinna* squid.

## Conclusion

Most previous observations have been of single *Magnapinna* squid, so the multiple sightings in the GAB have been quite revealing; whether they indicate a *Magnapinna* squid hotspot remains to be seen. These sightings, the first from Australian waters, have bolstered the hypothesis of a cosmopolitan distribution, and indicated a locally clustered distribution with squid being found in close spatial and temporal proximity of each other. In terms of morphology: measurements using paired lasers were provided for the first time rather than comparative scaling using objects of known size; *Magnapinna* squid in the size range of juveniles were confirmed as possessing long distal filaments, which are mostly missing from juvenile specimens due to damage; and in-situ colours were described in detail with distal filaments consistently white compared to the brown, orange, and pinks hues of the proximal arms/tentacles, mantle, and fins. Locomotive and behavioural components were observed, including a ‘raised arm’ posture, a horizontal example of the ‘elbow’ pose, and filament coiling, a behaviour never before seen in squids. The morphological, behavioural, and ecological insights gained from these *Magnapinna* sp. sightings reinforces the value of imagery as a tool in deep-sea squid research, and add to our knowledge of this elusive and intriguing genus.

## Supporting information

S1 Video Sighting 1 of *Magnapinna* sp. in the GAB. This video was taken by towed camera on 15 November 2015 at a depth of approximately 2178 m. The squid is first seen at approximately 3 seconds at the top middle of the field of view.
(MP4)

S2 Video Sighting 2 of *Magnapinna* sp. in the GAB. This video was taken by towed camera on 16 November 2015 at a depth of approximately 2110 m. The small *Magnapinna* squid (62 mm DML) can be seen from 6 seconds to 10 seconds into the video, just to the right of the centre of the screen.
(MP4)

S3 Video Sighting 3 of *Magnapinna* sp. in the GAB. This video was taken by ROV on 24 March 2017 at a depth of approximately 3060 m. Blue boxes are present in the lower corners to mask embedded logos in accordance with publishing requirements.
(MP4)

S4 Video Sighting 4 of *Magnapinna* sp. in the GAB. This video was taken by ROV on 25 March 2017 at a depth of approximately 3002 m. Blue boxes are present in the lower corners to mask embedded logos in accordance with publishing requirements.
(MP4)

S5 Video Sighting 5 of *Magnapinna* sp. in the GAB. This video was taken by ROV on 25 March 2017 at a depth of approximately 3056 m. Blue boxes are present in the lower corners to mask embedded logos in accordance with publishing requirements.
(MP4)
